# Simvastatin to modify neutrophil function in older patients with septic pneumonia (SNOOPI): study protocol for a randomised placebo-controlled trial

**DOI:** 10.1186/1745-6215-15-332

**Published:** 2014-08-22

**Authors:** Hannah Greenwood, Jaimin Patel, Rahul Mahida, Qian Wang, Dhruv Parekh, Rachel CA Dancer, Heena Khiroya, Elizabeth Sapey, David R Thickett

**Affiliations:** College of Medical and Dental Sciences, University of Birmingham, Vincent Drive, Birmingham, B15 2TT UK

**Keywords:** Neutrophils, Sepsis, Pneumonia, Simvastatin, Healthy Ageing

## Abstract

**Background:**

Community-acquired pneumonia (CAP) is considered the leading cause of death from infectious disease in developed countries, while complications of CAP - sepsis being the most common and challenging - increase the risk of mortality. During the progression of sepsis, a state of neutrophil ‘paralysis’ develops resulting in the impairment of neutrophil anti-microbial functions including: chemotaxis, production of reactive oxygen species, and formation of neutrophil extracellular traps (NETs). Mechanisms underlying defective neutrophil function remain elusive although NET formation has been implicated in the immunosuppression and increased rates of sepsis observed in neonates. There is, however, increasing evidence that statins are able to modulate neutrophil function in sepsis as several systematic reviews have concluded that statins have a role in improving infection-related outcomes and mortality while, *in vitro*, statins have also been shown to boost NET formation in healthy individuals.

**Methods/design:**

The ‘SNOOPI’ trial is a phase 4, randomised placebo-controlled trial. The aim of this study is to determine whether oral treatment with simvastatin compared to placebo optimises neutrophil anti-microbial functions in elderly patients with septic pneumonia improving patient outcomes in the elderly. The primary outcome will be NET production within 72 to 96 hours of treatment with simvastatin or placebo measured in response to a number of inflammatory mediators, including IL8, f-Met-Leu-Phe and lipopolysaccharide. Secondary outcomes include neutrophil migratory capacity; reactive oxygen species production; neutrophil phagocytic capacity; safety and tolerability of simvastatin administration within this patient group; biological markers of neutrophil activation, the inflammatory response, alveolar epithelial and endothelial injury; systemic endothelial function biomarkers and pulmonary extracellular matrix degradation. This study aims to recruit 60 patients admitted into Queen Elizabeth Hospital Birmingham NHS-Foundation Trust.

**Discussion:**

This study will investigate the ability of *in vivo* simvastatin therapy to modulate neutrophil anti-microbial functions in CAP-associated sepsis.

**Trial registration:**

EudraCT number: 2012-003343-29 (Trial Registered: 26 November 2012).

**Electronic supplementary material:**

The online version of this article (doi:10.1186/1745-6215-15-332) contains supplementary material, which is available to authorized users.

## Background

Community-acquired pneumonia (CAP) is considered the leading cause of death from infectious disease in developed countries. Increased mortality is associated with systemic manifestations and complications of CAP, with sepsis being the most common and challenging. The incidence of sepsis increases with age, while age is also a predictor of outcome in patients with pneumonia [1].

Sepsis is characterised by a state of systemic immune dysregulation and coagulopathy produced by invasive microorganisms and/or their microbial products, such as lipopolysaccharide (LPS). The initial cytokine storm and systemic inflammatory response syndrome (SIRS) is followed by a compensatory anti-inflammatory response which leads to a state of significant immuno-paralysis. This has been implicated in the progression of sepsis and subsequent development of acute lung injury (ALI), multi-organ dysfunction (MODS) and death [2, 3].

Neutrophils constitute our main immune defence against microbial infection by rapidly migrating to sites of infection and/or inflammation. In a process known as chemotaxis, neutrophils are able to sense and subsequently respond to shallow gradients of chemoattractants formed of chemotactic peptides, including cytokines, bacterial products and complement proteins [4]. Once at the site of infection, neutrophils act to eliminate any foreign material in an effort to prevent widespread tissue damage and ultimately resolve infection. During the progression of sepsis, a state of neutrophil ‘paralysis’ develops resulting in the impairment of neutrophil anti-microbial functions including: chemotaxis, production of reactive oxygen species (ROS), and formation of neutrophil extracellular traps (NETs) [5]. Unable to contain the infection, widespread dissemination of pathogenic material occurs throughout the host, augmenting inflammation and leading to the development of ALI and MODS [6]. Secondary to the development of ALI and MODS, this immune paralysis or chronic immunosuppression leads to an increased risk of secondary infections further exacerbating the increased risk of mortality.

Mechanisms underlying defective neutrophil function remain elusive although NET formation has been implicated in the immunosuppression and increased rates of sepsis seen in neonates [7], while other studies suggest NETs may exaggerate endothelial damage [8, 9]. The term NETs refers to the formation of extracellular fibres composed of de-condensed chromatin laden with anti-microbial proteins. Although phenotypically and functionally distinct from apoptosis, ‘NETosis’ does result in cell death; however, this allows neutrophils to exert their anti-microbial functions beyond their typical lifespan [10].

To date, there are no studies that specifically look at NET formation in CAP-associated sepsis. There is, however, increasing evidence that statins are able to modulate neutrophil function in sepsis with a recent study demonstrating the ability of statins to reduce the neutrophil infiltrate following LPS challenge in bronchoalveolar lavage fluid taken from patients receiving simvastatin prior to challenge [11]. Furthermore, the reduction in neutrophil counts was not associated with a decrease in pro-inflammatory mediator expression, suggesting it reflects a reduced neutrophil response or increased neutrophil apoptosis [11]. Statins inhibit 3-hydroxy-3 methylglutaryl coenzyme A reductase, a rate-limiting enzyme in the biosynthesis of cholesterol, and have been shown to exert numerous effects in addition to their lipid-lowering properties including potentially anti-inflammatory and immunomodulatory effects [1]. Several systematic reviews have concluded that statins have a role in improving infection-related outcomes and mortality; however, most of this evidence is limited to retrospective and prospective observational studies [12, 13]. *In vitro*, statins have also been shown to boost NET formation in healthy individuals with the consequent elimination of bacteria - an effect which could therefore be an important determinant of outcome in patients with CAP-associated sepsis [14].

### Impaired neutrophil chemotaxis in the elderly

Neutrophils from healthy elderly donors demonstrate features of immune-paralysis that resemble those seen in patients with sepsis: healthy elderly patients (over 60 years) demonstrate defective directional migration (chemotaxis) and overall accuracy (chemotactic index) whilst maintaining their speed of migration (chemokinesis) when migrating towards IL8 and the bacterial protein f-Met-Leu-Phe (fMLP) [15].

### Statins and sepsis

The ASEPSIS trial, a phase II randomised controlled trial, demonstrated that acute administration of atorvastatin reduces the progression of sepsis to severe sepsis [16]. These changes were not associated with systemic levels of cytokines or chemokines in contrast to other reported studies suggesting the cellular effects of statins may be more important than modulation of systemic mediators.

### Community-acquired pneumonia-associated sepsis and neutrophil extracellular traps

During the early stages of CAP-associated sepsis, NET production is suppressed with improvements occurring during resolution. In addition, NET release was significantly lower in CAP patients who meet the criteria for severe sepsis. Taken together these data suggest that neutrophil function is a feature of CAP-associated sepsis, especially in the elderly, and that statin therapy may have potential to restore the defects in these neutrophils both *in vivo* and *in vitro*.

In light of these data, we hypothesise that the increased incidence and poorer outcomes observed in the elderly population in relation to pneumonia and sepsis may be due to defective neutrophil function, and that restoration of these functions may therefore improve outcome, especially in the elderly. The SNOOPI trial therefore aims to determine whether oral treatment with simvastatin optimises neutrophil anti-microbial functions in elderly patients admitted into Queen Elizabeth Hospital Birmingham (QEHB) with septic pneumonia, improving patient outcomes in the elderly. Alterations in neutrophil function will be related to clinical outcome, disease-relevant severity scores and biomarkers present in blood and urine.

## Methods/design

### Trial approvals and conduct

The trial is approved by Yorkshire and Humber Ethics Committee (REC 12/YH/0375) and is registered on the European Clinical Trials Database (EudraCT Number: 2012-003343-29). The trial sponsor is the University of Birmingham (Reference: RG_12-179). The trial is funded by the British Lung Foundation (BLF reference LT/12) and will be carried out in accordance with the principles of the International Conference on Harmonisation Good Clinical Practice guidelines, applicable UK legislation and standard operating procedures of the Respiratory Research Group at the University of Birmingham. Results of the trial will be reported in accordance with consolidated Standards of Reporting Trials 2010 guidelines [17].

### Outcome measures

#### Primary outcome

The primary outcome will be NET production within 72 to 96 hours of treatment with simvastatin or placebo, measured in response to a number of inflammatory mediators including IL8, fMLP and LPS and using phorbol-12-myristate-13-acetate as a positive control [18] (for full methods, see Additional file 1).

#### Secondary outcomes

Secondary outcomes of this trial include parameters describing neutrophil migratory capacity; ROS production; neutrophil phagocytic capacity; safety and tolerability of simvastatin administration within this patient group; biological markers of neutrophil activation (for example, myeloperoxidase), the inflammatory response (for example, IL6), alveolar epithelial and endothelial injury (for example, von Willebrand factor), and systemic endothelial function biomarkers (for example, albumin:creatinine ratio); pulmonary extracellular matrix (ECM) degradation and turnover of peptide; and, finally, the relationship of baseline and changes in parameters described above to clinical relevant outcomes (survival, development of organ failure, admission onto intensive therapy unit, sequential organ failure assessment score, ventilator-free days and muscle wastage during the inpatient stay). These assessments will be made at baseline, on days 4 and 7, and in convalescent samples.

Neutrophil migratory capacity will be measured using a modified Dunn chamber as described previously [15, 19]; neutrophil ROS production will be measured using a luminol-based assay and phagocytic capacity measured by flow cytometry. Plasma indices of neutrophil activation, the inflammatory response and alveolar epithelial and endothelial injury will be measured by enzyme-linked immunosorbent assay, and systemic endothelial function biomarkers and pulmonary ECM degradation will be measured in urine collected from an indwelling urinary catheter (for full methods, see Additional file 1). These assessments will be made at baseline, on days 4 and 7, and in convalescent samples (≥6 weeks post-discharge).

### Eligibility criteria

Patients will be eligible for the trial if they fulfil the following criteria:

- Aged over 60 years.
- Meet the British Thoracic Society guidelines for diagnosis of CAP, namely symptoms and signs consistent with an acute lower respiratory tract infection associated with radiographic shadowing for which there is no other explanation [20]. Signs and symptoms include three or more of the following: cough, sputum production, breathlessness, pleuritic chest pain, haemoptysis, fever, headache, signs consistent with pneumonia on chest auscultation.
- CAP patients will also need to meet the criteria for sepsis based on the standard definitions as published in the 2008 Surviving Sepsis Campaign Guidelines [21] as illustrated in Table  1.
- All sepsis and pneumonia criteria must occur within the same 24-hour period (sepsis is defined as evidence of infection plus SIRS; see Table 2).
- Patients must be enrolled within 48 hours of admission to hospital.

**Table 1:** **Surviving Sepsis Campaign Guideline definitions of sepsis**

Condition	Definition
Sepsis	SIRS plus new-suspected infection
Severe sepsis	Sepsis plus sepsis-induced organ dysfunction
Organ dysfunction	Sepsis-induced hypotension
	Lactate > normal laboratory results
	Urine output <0.5 mL/kg/hour for >2 hours despite adequate fluid resuscitation
	ALI with PaO_2_/FiO_2_ < 250 in the absence of pneumonia as infection source
	ALI with PaO_2_/FiO_2_ < 200 in the presence of pneumonia as infection source
	Creatinine >176.8 mmol/L
	Bilirubin >34.2 mmol/L
	Platelet count >100,000/mm^3^
	Coagulopathy (INR >1.5)
Septic shock	Severe sepsis plus hypotension not reversed by fluid resuscitation

ALI, acute lung injury; FiO_2_, fraction of inspired oxygen; INR, International normalised ratio; PaO_2_, partial pressure of oxygen in blood; SIRS, systemic inflammatory response syndrome.

**Table 2:** **Systemic inflammatory response syndrome diagnostic criteria**

	Criteria:
Two or more of the following:	Fever of ≥38°C or ≤36°C
	Heart rate ≥90 beats per minute
	Respiratory rate ≥20 breaths per minute or PaCO_2_ levels ≥32 mmHg
	Abnormal white blood cell count (>12,000/μl or <4,000/μl or >10% bands)
	PaCO_2_, partial pressure of carbon dioxide in blood.

Patients fulfilling any of the below criteria will be excluded from the study:

- More than 48 hours from admission at time of consent
- Current or recent statin use within 1 month.
- Known prior myositis.
- Creatinine kinase >10 times upper limit of normal range*.
- Transaminases (alanine aminotransferase/aspartate aminotransferase) >8 times upper limit of normal range*.
- Severe renal impairment (creatinine clearance <30 ml/min) not receiving renal replacement therapy.
- Patients currently receiving ongoing and sustained treatment with any of the following: itraconazole, ketoconazole, HIV protease inhibitors, nefazodone, ciclosporin, amiodarone, verapamil or diltiazem, Fibric acid derivatives (except fenofibrate), danazol.
- A family history of muscular disorders.
- Known HIV or hepatitis B/C infection.
- Contraindication to enteral drug administration (either orally or via nasogastic tube; for example, patients with mechanical bowel obstruction).
- Known participation in other investigational medicinal product (IMP) trials within 30 days.
- Consent/relative or advocate assent declined.
- Treatment withdrawal imminent within 24 hours.
- Non-English speaking patients or those who do not adequately understand verbal or written information unless an interpreter is available.
- Immunosuppression due to corticosteroid or other immunosuppressant use.

(*If creatinine kinase, alanine aminotransferase or aspartate aminotransferase values are not available as part of routine care, a blood sample will be obtained after informed consent but before randomisation. Creatinine kinase, alanine aminotransferase and aspartate aminotransferase values may be obtained up to 48 hours prior to randomisation).

### Power and sample size estimate

Mean NET production in CAP-associated sepsis patients is suppressed down to 7,500 arbitrary units (AU) versus 13,200 (SD 2,432) AU in age-matched control neutrophils. At day 4, levels remain suppressed at 8,200 AU (SD 5,100) but, by day 7, patient neutrophils have increased baseline NET production almost back to normal (11,500 AU (SD 6,100), *P* = 0.9) versus normal matched controls. If we assume that statin treatment *in vivo* has a similar magnitude of effect as it does *in vitro* on elderly patient neutrophils (+80% NET production), then 18 patients in each arm would be needed to increase NET production by 5,000 AU from day 0 to day 4 with a power of 0.8, *P* = 0.05. For 90% power we would need 23 patients in each arm. To allow for drop outs due to death and so forth, we intend to recruit 30 patients in each arm. Based on our other preliminary data with 23 patients completing each arm, we would have an 80% power to detect significant increases in both chemotactic velocity (effect size +0.5, ±35%) and ROS production (effect size ±57%). For *in vitro* work on healthy elderly patient neutrophils we typically require 6 to 8 patient samples for mechanistic studies so these patient numbers should be adequate for additional mechanistic work.

### Trial conduct

#### Approach to patients and obtaining informed consent

Patients will be identified on the daily consultant ward rounds that take place on the 40-bed clinical decision unit and 80-bed ICU at the QEHB (approximately 60 to 70 daily admissions). Eligible patients may only be included in the trial after obtaining written informed consent.

Where necessary, informed consent may be sought from the patient’s personal legal representative who may be a relative or close friend. If the patient is unable to give informed consent and no personal legal representative is immediately available, a senior doctor (consultant or registrar) who is not connected with the trial may act as professional legal representative.

A copy of the signed informed consent form will be placed in the patients’ medical records and the originals will be retained by the personal legal representative and by the Principal Investigator in the investigator site file.

#### Randomisation and drug/placebo supply

The trial drug (Simvastatin, 80 mg; trade name Accord) and matching placebo will be manufactured by Sharp Clinical Services Ltd (Crickhowell, UK) according to principles of good manufacturing pratice. Matching IMPs will be supplied and packaged according to the randomisation sequence by Sharpe Clinical Services and delivered to the QEHB pharmacy. Patients will be randomised sequentially by allocating them to the next numbered treatment pack held in the QEHB pharmacy.

#### Administration of investigational medicinal product

Subjects will receive either 80 mg simvastatin or placebo once daily for 7 days or until hospital discharge, whichever is shorter. The first dose of study drug will be administered as soon as possible, ideally within 4 hours of randomisation and all subsequent doses given each morning starting on the following calendar day. The drug will be administered by qualified medical or nursing staff. If patients exceed the liver function test, renal or creatinine kinase entry criteria during drug treatment, IMP administration will be withdrawn.

#### Concomitant medications

Patients taking the following medications are not eligible for participation in the study:

- Current or recent statin use within 1 month.
- Taking itraconazole, ketoconazole, HIV protease inhibitors, nefazodone, ciclosporin, amiodarone, verapamil or diltiazem, fibric acid derivatives (except fenofibrate) or danazol.

Severely ill pneumonia patients are usually treated with a combination of intravenous penicillin and a macrolide antibiotic, typically clarithromycin 500 mg twice a day in our trust. If we were to exclude patients who were taking clarithromycin, the trial would therefore only be able to enrol patients with mild disease or those known to be intolerant of macrolides. This would be a significant barrier to recruitment and limit the generalizability of any finding of the study to the general population. We will therefore not exclude patients who are receiving concomitant clarithromycin or erythromycin from this study.

#### Post-randomisation withdrawals and exclusions

Patients may withdraw or be withdrawn (by the personal legal representative or professional legal representative) from the trial at any time without prejudice. Data recorded up to the point of withdrawal will be included in the trial analysis unless consent to use their data has also been withdrawn. If a patient or legal representative withdraws consent during trial treatment, the trial drug will be stopped but permission will be sought to access medical records to collect data related to the trail as per the protocol until the end of the trial.

#### Blinding/unblinding

All personnel involved in the trial, including patients, clinical staff and research/trial staff, will be unaware of the arm of the study to which the patient is allocated during trial participation. Active and placebo treatment packs of the IMP will be identical in packaging, appearance and contents. The protocol allows for emergency unblinding in the event of serious concerns about patient safety. In the unlikely event that unblinding is required the local investigator will discuss this with the chief investigator. All events will be logged.

#### Monitoring and reporting adverse events

All adverse events and serious adverse events, irrespective of the causal relationship with the trial medications, will be reported to the chief investigator within 24 hours of awareness by the investigator. The chief investigator will inform the sponsor and regulatory authorities. As the IMPs used in this study are licensed in the UK, the expected serious adverse reactions (SARs) will be recorded on the case report form.

#### Data collection

Data will be collected on individual case report forms, designed and utilised by the research team to record patient data until hospital discharge. Submitted data will be anonymised and data stored securely against unauthorised access and accidental loss. Data collected will include demographic details, as well as physiological and ventilator variables that will act as indicators of disease severity. If the subject remains in hospital at 28 or 90 days, survival at these time points will be recorded by research staff.

#### Statistical analysis plan

Data will be analysed with the help of a trial statistician. Data will be analysed using SPSS for windows 17.0 (IBM, NY, USA). A detailed analysis plan will be developed during the trial prior to commencement of analysis. In brief, for continuously distributed data, differences between groups will be tested using independent samples *t*-tests with transformations of variables to normality if appropriate or non-parametric equivalents. Chi-squared tests (or Fishers Exact tests) will be used for categorical variables. A *P* value of 0.05 will be considered as significant. We will test for significant correlations between changes in the biological markers using standard methods. The treatment effect will be analysed on an intention-to-treat basis. For the further examination of relationships between a binary variable and known explanatory variables, the following tests will be applied as appropriate. Logistic regression will be used to provide the estimated risk ratios for the treatment effect with associated 95% confidence intervals. Time to event outcomes such as duration of ventilation or duration of hospital stay will be analysed by survival methods and reported as hazard ratios and 95% confidence intervals. A single final analysis is planned at the end of the trial.

#### Trial organisation/oversight

Trial oversight will be provided by a Trial Steering Committee comprising investigators, clinicians and trialists. An independent data monitoring committee will monitor the safety of participants enrolled in the trial through regular review of adverse event reports. An interim analysis of efficacy is not planned.

## Discussion

Preliminary data on which this study is based suggests a significant role of neutrophil immune-paralysis in the development and progression of sepsis, a process that may be amenable to correction by simvastatin. This study will investigate the ability of *in vivo* simvastatin therapy to modulate neutrophil anti-microbial functions in CAP-associated sepsis and ultimately improve patient outcomes.

## Trial status

Patient recruitment commenced in December 2013 and is expected to continue for 18 months with patient recruitment ending in April 2015.

## Electronic supplementary material

Additional file 1: **Methods.** Full methods for analysis of primary and secondary outcomes. (DOCX 23 KB)

## Abbreviations

ALI: acute lung injury
CAP: community-acquired pneumonia
ECM: extracellular matrix
fMLP: f-Met-Leu-Phe
IL: interleukin
IMP: investigational medicinal product
LPS: lipopolysaccharide
MODS: multi-organ dysfunction
NET: neutrophil extracellular trap
QEHB: Queen Elizabeth Hospital Birmingham
ROS: reactive oxygen species
SIRS: systemic inflammatory response syndrome.
