# Supplementary material for: Simvastatin to modify neutrophil function in older patients with septic pneumonia (SNOOPI): study protocol for a randomised placebo-controlled trial
Source: Trials. 2014 Aug 22;15:332. doi: 10.1186/1745-6215-15-332 (PMC4247744; doi:10.1186/1745-6215-15-332)
Supplement: Supplementary file 1 — Additional file 1: Methods. Full methods for analysis of primary and secondary outcomes. (DOCX 23 KB) [file 13063_2014_2315_MOESM1_ESM.docx]

Online supplement:

**Simvastatin to Modify Neutrophil Function in Older Patients with Septic Pneumonia (SNOOPI): study protocol for a randomised placebo controlled trial**

Hannah Greenwood (h.l.greenwood@bham.ac.uk)^1^,

Jaimin Patel (Jaimin.patel@doctors.org.uk)^1^,

Rahul Mahida (r.mahida@bham.ac.uk)^1^,

Qian Wang (wangqian84@aliyun.com)^1^,

Dhruv Parekh (d.parekh@bham.ac.uk)^1^,

Rachel C A Dancer (r.dancer@bham.ac.uk)^1^,

Heena Khiroya (heenakhiroya@doctors.org.uk)^1^,

Elizabeth Sapey (lizsapey@aol.com)^1^,

David R Thickett (d.thickett@bham.ac.uk)^1, 2^

^1^College of Medical and Dental Sciences, University of Birmingham, Vincent Drive, Birmingham B15 2TT

^2^Corresponding Author: [d.thickett@bham.ac.uk](mailto:d.thickett@bham.ac.uk)

**Laboratory Methods:**

*Storage of Plasma, Serum and Urine*

Serum and plasma samples will be centrifuged (560 x g, 10 minutes) to remove any contaminating cells and then stored at -80°C in -0.5ml aliquots until use. All outcomes relating to stored serum and plasma samples will be measured simultaneously once trial recruitment is complete.

*Neutrophil Isolation*

Whole blood will be drawn from patients admitted into QEHB with septic pneumonia by venepuncture carried out by qualified phlebotomist. Neutrophils will be isolated from peripheral blood anti-coagulated with either lithium heparin for quantification of NETs and apoptosis or Ethylenediaminetetraacetic Acid (EDTA; BD Biosciences, UK) for quantification of migratory capacity and phagocytosis. Neutrophils will be isolated by separation on a Percoll density gradient (pH 8.5-9.5; Sigma Aldrich, UK) as described previously ([1](#_ENREF_1)). Following isolation, neutrophils will be re-suspended in sterile RPMI-1640 (with sodium bicarbonate and L-glutamine, Sigma Aldrich) supplemented with GPS (Sigma Aldrich, UK) at cell densities relevant for use in functional assays. Sample will be assessed using a commercial Giemsa statining kit (Reastain Quick-Diff Kit, Gentaur Europe) and analysis carried if purities exceed ≥ 95% neutrophils and viability above 98% as assessed by trypan blue exclusion. Where appropriate, neutrophils will be primed by incubation with 10ng/ml tumour necrosis factor-α (TNFα) for 15 minutes at 37°C in a humidified 5% CO_2_ atmosphere.

*Quantification of neutrophil extracellular trap (NET) production*

Freshly isolated neutrophils will be re-suspended at 1x10^6^/ml in RPMI-1640 supplemented with GPS (Sigma Aldrich, UK). NET production will be measured as described previously ([2](#_ENREF_2)). Briefly, resting and primed neutrophils will be incubated with 25nM phorbol 12-myristate (PMA) (Sigma Aldrich, UK), 1.25nM IL8 (R&D Systems, UK), 100ng/ml lipopolysaccharide (LPS) (Sigma Aldrich, UK) or 2.5μM fMLP (Sigma Aldrich, UK) for 3 hours at 37°C in a humidified 5% CO_2_ atmosphere. Following incubation, extracellular DNA will be digested and stained using 1U/ml-1 micrococcal nuclease (MNase, Sigma Aldrich, UK) and 1μM SYTOX Green (Life Technologies, UK) respectively by incubation in the dark at room temperature. Cells will then be pelleted and DNA content of the supernatant quantified by measuring fluorescence using a BioTek Synergy 2 flouometric plate reader (NorthStar Scientific Ltd, Leeds, UK excitation at 485nm and emission at 530nm). All experiments will be performed in quadruplicate and background fluorescence subtracted from test samples.

*Neutrophil Migration*

Freshly isolated neutrophils will be re-suspended at 2x10^6^/ml in RPMI-1640 supplemented with GPS (Sigma Aldrich, UK). Neutrophil Migratory dynamics will be measured using a modified Dunn chamber as described previously ([3](#_ENREF_3)). Migration will be quantified using 3 parameters: Chemokinesis (speed of migration), Chemotaxis (directional migration) and Chemotactic Index (accuracy of migration) relevant to a chemotactic gradient made up of either interleukin-8 (IL-8) or the bacterial protein f-Met-Leu-Phe (fMLP) as described previously ([3](#_ENREF_3)).

*Neutrophil Phagocytosis*

Freshly isolated neutrophils will be re-suspended at 1x10^6^/ml in RPMI-1640 containing GPS and phagocytosis quantified using a commercially available assay utilizing *Staphylococcus aureus* and *Escherichia coli* pHrodo labelled bio particles (Invitrogen Life Technologies, UK) according to the manufacturer’s instructions ([4](#_ENREF_4), [5](#_ENREF_5)). Phagocytosis will be quantified by flow cytometry.

*Neutrophil Apoptosis*

Neutrophil apoptosis will be measured immediately following isolation and again at 4 hours and 24 hours post isolation using a commercially available apoptosis detection kit (PE Annexin V Apoptosis Detection Kit I, BD Biosciences, UK) with levels of apoptosis being quantified by flow cytometry ([6](#_ENREF_6)).

*Neutrophil Reactive Oxygen Species (ROS) production*

Production of reactive oxygen species will be measured using a luminol-based chemiluminescence on freshly isolated neutrophils re-suspended at 1x10^6^/ml in hank’s buffered salt solution containing 12.61mM CaCl_2_, 4.93mM MgCl_2_, 4.07mM MgSO_4_ and phenol red free (HBSS^+/+^, Gibco Invitrogen, UK) as described previously ([2](#_ENREF_2)). Briefly, freshly isolated neutrophils will be stimulated with 100ng/ml LPS (Sigma Aldrich, UK), 10ng/ml IL8 (R&D Systems, UK) or 25nM PMA (Sigma Aldrich, UK) in the presence of 1μM luminol (pH 7.3, Sigma Aldrich, UK). ROS generation will be measured a 1 minute intervals using a Berthold Centro LB 960 luminometer (Berthold Technologies, Hertfordshire, UK) as relative light units and will be presented as area under the curve (AUC). All experiments will be performed in quadruplicate.

*Quantification of markers of neutrophil activation, the inflammatory response and alveolar epithelial & endothelial injury*

Markers of neutrophil activation, the inflammatory response and alveolar epithelial and endothelial injury will be measured simultaneously by ELISA once trial recruitment is complete. ELISA kits will be sourced from R&D Systems e.g. TNFα, IL-6, soluble ICAM (sICAM) (R&D Systems, Abingdon, UK)

**Supplementary References**

1. Butcher SK, Chahal H, Nayak L, Sinclair A, Henriquez NV, Sapey E, et al. Senescence in innate immune responses: reduced neutrophil phagocytic capacity and CD16 expression in elderly humans. Journal of leukocyte biology. 2001;70(6):881-6. Epub 2001/12/12.

2. Hazeldine J, Harris P, Chapple IL, Grant M, Greenwood H, Livesey A, et al. Impaired neutrophil extracellular trap formation: a novel defect in the innate immune system of aged individuals. Aging cell. 2014. Epub 2014/05/02.

3. Sapey E, Greenwood H, Walton G, Mann E, Love A, Aaronson N, et al. Phosphoinositide 3-kinase inhibition restores neutrophil accuracy in the elderly: toward targeted treatments for immunosenescence. Blood. 2014;123(2):239-48. Epub 2013/11/06.

4. Invitrogen. pHrodo Red *S.aureus* Bioparticles Conjugate for Phagocytosis. Available from: http://www.lifetechnologies.com/order/catalog/product/A10010.

5. Invitrogen. pHrodo Red *E.coli* BioParticles Conjugate for Phagocytosis. Available from: http://www.lifetechnologies.com/order/catalog/product/P35361.

6. BD-Biosciences. Annexin V : PE Apoptosis Detection Kit I. Available from: https://www.bdbiosciences.com/ptProduct.jsp?prodId=13189&key=559763&param=search&mterms=true&from=dTable.
